# Impact of Cusp-Overlap View for TAVR with Self-Expandable Valves on 30-Day Conduction Disturbances

**DOI:** 10.1155/2021/9991528

**Published:** 2021-04-28

**Authors:** Oscar A. Mendiz, Marko Noč, Carlos M. Fava, Luis Abel Gutiérrez Jaikel, Matias Sztejfman, Aleš Pleskovič, Paul Gamboa, León R. Valdivieso, Hemal Gada, Gilbert H. L. Tang

**Affiliations:** ^1^Cardiology and Cardiovascular Surgery Institute (ICyCC), Favaloro Foundation University Hospital, Buenos Aires, Argentina; ^2^MC Medicor, International Center for Cardiovascular Diseases, Izola, Slovenia; ^3^Interventional Cardiology, Hospital Clínica Bíblica, San José, Costa Rica; ^4^Interventional Cardiology Department, Sanatorio Finochietto, Buenos Aires, Argentina; ^5^UPMC Heart and Vascular Institute, Pinnacle Health, Harrisburg, PA, USA; ^6^Department of Cardiovascular Surgery, Mount Sinai Health System, New York, NY, USA

## Abstract

**Methods and Results:**

We retrospectively compared 257 consecutive patients undergoing TAVR with self-expandable valves using either CON (*n* = 101) or COVL (*n* = 156) in four intermediate/low volume centers. There were no significant differences in baseline characteristics between the groups. The 30-day incidence of new-onset LBBB (12.9% vs. 5.8%; *p*=0.05) and PPMI rate (17.8% vs. 6.4%; *p*=0.004) was significantly lower when using the COVL implantation view. There was no difference between the CON and COVL groups in 30-day incidence of death (4.9% vs. 2.6%), any stroke (0% vs. 0.6%), and the need for surgical aortic valve replacement (0% for both groups).

**Conclusion:**

Using the COVL view for implantation, we achieved a significant reduction of the LBBB and PPMI rate after TAVR in comparison with the traditional CON view, without compromising the TAVR outcomes when using self-expandable prostheses.

## 1. Introduction

Transcatheter aortic valve replacement (TAVR) is recommended for intermediate and high-risk surgical patients with severe aortic stenosis, and new evidence is now also supporting its use for low-risk patients. Although periprocedural complications have been decreasing over time, conduction disturbances leading to permanent pacemaker implantation (PPMI) remains a common complication which has been related to a higher 1-year mortality. This complication would be even more relevant in low-risk and younger patients receiving TAVR [1]. Although conduction disturbances requiring PPMI after TAVR even for self-expandable valves have been decreasing with newer devices, operator's experience and new deployment techniques still remain as a significant limitation which should be addressed [2]. In this study, we compared 30-day rates of new-onset persistent LBBB and PPMI rates between patients undergoing TAVR with the self-expandable Evolut R™ and Evolut PRO™ (Medtronic) transcatheter heart valves (THV) using the conventional 3-cusp coplanar implantation view (CON) and the recently described right/left cusp-overlap view (COVL), with the hypothesis that this new strategy can significantly reduce the PPMI rate after TAVR.

## 2. Methods

From August 2019 to June 2020, 156 consecutive patients underwent elective or emergent TAVR using right/left COVL view with self-expandable THV in three intermediate/low volume centers from Latin-America and one European center. These patients were retrospectively compared to 101 consecutive patients who underwent TAVR immediately before August 2019 using the CON view with the same self-expandable THV. According to each local heart team, all patients were considered as high surgical risk candidate. Patients treated during this period of time with a bicuspid aortic valve (13), having previous implanted permanent pacemaker (*n* = 25), receiving balloon-expandable valve implantation (*n* = 76) or other self-expandable valves (Acurate Neo™ (*n* = 32) and Portico™ (*n* = 19)) and those who underwent a valve-in-valve procedure (21) were excluded.

All study patients underwent transthoracic echocardiography and multidetector computed tomography to evaluate aortic root dimensions, morphology, and calcification.

### 2.1. Transcatheter Heart Valve Implantation Technique

Prostheses were sized using manufacturer recommendations, including annular and LVOT dimensions and location and severity of annular and LVOT calcification.

**Figure 1:**
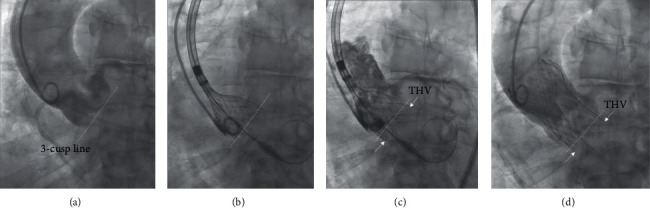
THV implantation in conventional 3-cusp coplanar view. (a) 3-cusp coplanar view (CON) in LAO projection. (b, c) THV positioning in the CON view. (d) Final angiographic outcome after using a standard 3-cusp coplanar projection (29 mm Evolute R™ THV). LAO = left anterior oblique, THV = transcatheter heart valve, CON = 3-cusp coplanar view.

In all patients in the CON group (Figure 1), transcatheter heart valve (THV) positioning and implantation was done using a standard 3-cusp coplanar projection, which was selected based on previous CT scan evaluation and corrected under angiographic view before starting the procedure, usually in the left anterior oblique view. After crossing the aortic valve with the nose cone of the delivery catheter, it was centering, and deployment was started at annulus level or no further than 1 mm below. The THV parallax was eliminated using a more cranial or caudal angulation. We guided the positioning following the point of contact between THV and noncoronary cusp up to the contact point with the left-coronary cusp while keeping tension or gently pulling the delivery system to prevent THV protrusion into the left ventricle outflow tract. After initial positioning of the THV, any correction or repositioning were done according to the operators' discretion with the intention to achieve an implantation 2 to 4 mm below the annulus. Rapid pacing during valve deployment was used at the operators' discretion.

**Figure 2:**
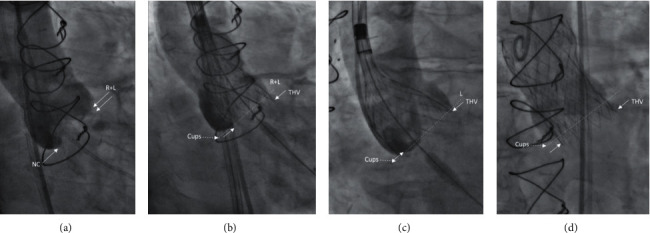
THV implantation in the right and left cusp-overlap view. (a) COVL view (right and left cusps overlap in the RAO caudal view). (b) THV positioning in the COVL view. (c) 3-cusp conventional view during positioning (LAO cranial) where THV locks higher than the COVL view. (d) After final delivery in the COVL view (34 mm Evolute R™ THV). COVL = cusp-overlap view, RAO = right anterior oblique, THV = transcatheter heart valve, LAO = left anterior oblique.

For the COVL strategy [3] (Figure 2), all patients have a previous CT evaluation and right and left cusp-overlap projections were identified before the procedure during the CT image evaluation and validated by intraprocedural aortic root angiography at the COVL view. In some cases, when perfect overlapping was not easily obtained or there were contrast media restrictions, two pigtail catheters were positioned in the right and left cusps to assure optimal overlapping between them. THV placement was started in the middle of the pigtail loop, just above the annulus in order to minimize LVOT contact with the delivery system and allowing THV to dive into the left ventricle outflow tract up to 2 to 3 millimeters below the annulus. When the valve started flaring until the left cusp contact was reached, rapid pacing (120 bpm) was performed using either a temporary pacemaker in the right ventricle or THV delivery wire. Left cusp contact was confirmed on the left anterior oblique view, but final implantation depth was usually assessed in the COVL view, except for rare cases in which the left view was used. In case of repositioning, initial COVL was used to start the deployment all over again.

Balloon predilatation, always undersized, and postdilatation were performed at operators' discretion.

All patients had preprocedural 12-lead electrocardiogram which was repeated, after procedure, at 24 hours, predischarge, and 30-days after TAVR. PPMI was considered in patients with persistent complete A-V block and in those with preexisting conduction abnormalities including the right bundle branch block and first‐degree atrioventricular block who develop high‐grade atrioventricular block during or after THV deployment which did not spontaneously recover within 24 hours, or in cases without preexisting conduction disorder who developed high‐grade atrioventricular block after 24 hrs.

The study was conducted in accordance with the provisions of the Declaration of Helsinki and with the International Conference on Harmonization Good Clinical Practices, and all patients signed the regular hospital informed consent for the procedure.

### 2.2. Outcomes

Primary clinical outcomes were 30-day LBBB and PPMI rates. We also analyzed 30-day all-cause mortality, stroke, urgent surgery, coronary occlusion, and valve pop-out after final delivery.

Procedural outcomes were reported according to Valve Academic Research Consortium-2 definitions; 30-day major cardiac and cerebrovascular adverse events (MACCE) included 30-day all-cause death, surgical aortic valve replacement (SAVR), myocardial infarction, and any stroke. Vascular complications and bleeding were also defined according to Valve Academic Research Consortium-2 criteria [4, 5]

Data from the four-hospital series were obtained from clinical records and 30-day clinical outcomes via clinical visits or telephone contacts and merged. They included baseline demographics, medical history, baseline and postprocedural electrocardiograms, preprocedural CT scan evaluation, and procedural characteristics.

### 2.3. Statistical Analysis

Clinical, anatomic, CT scan measurements and procedural characteristics of patients using COVL implantation technique were compared with those using CON view.

Continuous variables are presented as mean ± standard deviation for variables following normal distribution, whereas nominal variables are presented as absolute values and percentages. Continuous variables between the groups were compared by Student's *t*-test and nominal by the Chi-square test or Fisher's exact test. All statistical tests were 2-tailed, with *p* values <0.05 considered to indicate statistical significance. All statistical analyses were performed using SAS version 9.3 (SAS Institute, Cary, North Carolina).

## 3. Results

Baseline population characteristics are shown in Table 1. Conscious sedation and femoral access ware used in all cases; in one case in the CON group, elective surgical cutdown was used. Percutaneous vascular access was used in all cases using Prostar XL™ and Proglide™ (Abbott Vascular, Redwood City, CA) closure devices. There were no valve pop-outs after final delivery for both groups.

THV implanted were Evolute R™ and Pro™ for all patients in both groups. Predilation and postdilation were used in 57.4% vs. 55.1% (*p*=0.18) and 24.7% vs. 21.8% (*p*=0.58) in the CON and COVL groups.

Prostheses were sized using manufacturer recommendations; the oversizing of THV to annulus perimeter was similar between CON and COVL groups: 19.2% vs. 19.6% (*p*=0.57).

Implantation success was achieved in all cases and clinical success, in 95.1% vs. 97.4% (*p*=0.30). Moderate paravalvular leak occurred in 2.0% and 2.5% (*p*=0.76), and there were no severe leaks in either group.

MACCE rates at 30 days were the following: any death: 4.9% vs. 2.6% (*p*=0.3); major stroke: 0% vs. 0.6% (*p*=0.42); MI: 0% vs. 0.6% (*p*=042); and there were no minor stroke or surgical aortic valve replacements.

**Figure 3:**
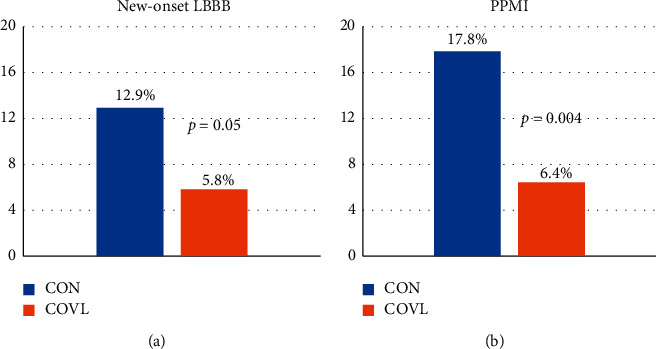
Conduction disturbances after TAVR using cusp-overlap view vs. conventional 3-cusp coplanar view. 30-day new-onset LBBB and permanent pacemaker implantation rate using cusp-overlap projection for TAVR in comparison with the standard 3-cusp coplanar projection view using self-expandable THV. LBBB = left bundle branch block; THV = transcatheter heart valve.

Primary clinical outcomes: new-onset LBBB appeared in 13 (12.9%) patients in the CON vs. 9 (5.8%) of the COVL group (*p*=0.05), while 30-day PPMI rate was 18 (17.8%) vs. 10 (6.4%) for CON and COVL groups (*p*=0.004) (Figure 3).

Vascular complications occurred in 2 (2%) vs. 6 (3.8%) (*p*=0.4) and major bleeding complications in 2 (2%) vs. 1 (0.6%) (*p*=0.32) for CON and COVL groups, while hospitalization time was similar, COVL group 2.8 ± 1.1 vs. 2.7 ± 1.1 days for each group (*p*=0.) (Table 2).

THV depth measure by angiography in the COVL view was available in 90% of the COVL group patients and was 3.43 ± 2.79 mm from the noncoronary cusp to the deepest end of THV in the LVOT and 5.65 ± 3.48 mm from the right/left overlapped cusps. These measurements were not available for most of the CON group because final angiography was usually performed in the LAO view.

## 4. Discussion

Increased operators' experience and THV design improvements have reduced the risk for TAVR-related complications; moreover, TAVR is rapidly expanding toward younger and lower-risk patients. In this scenario, although periprocedural PPMI rate after TAVR implantation has been decreasing over the past few years, conduction disturbances still remain as one of the most common complication which can also affect late follow-up [1, 2, 6–8].

PPMI after TAVR is necessary in 10% to 30% of patients depending on some previous anatomic conditions, the type of THV implanted, procedural features, and operators' experience and represents a significant limitation which should be especially considered when treating young patients [6–9].

New-onset persistent LBBB and PPM implantation have been associated with increased early and late all-cause mortality and a higher risk of heart failure rehospitalizations [10]. Thus, any improvement to prevent conduction disturbances would potentially reduce late mortality rate, rehospitalizations, and cost after TAVR [11, 12].

Different studies have confirmed a relationship between THV implantation depth in the LVOT and PPMI rate [2]. It is worth mentioning that other nonprocedural-related factors such as LVOT calcification are also predictive of PPM implantation, but not possible to be modified by operators during THV implantation [13–15].

Due to preexisting RBBB, balloon predilatation and self‐ and mechanically expanding valves use were identified as independent predictors of PPMI after TAVR [16]; many authors suggested not using these valves and not to use predilatation in patients with previous RBBB [17, 18].

The present study comparing the conventional 3-cusp view (CON) with recently introduced right and left cusp-overlap view (COVL) for self-expandable valve implantation demonstrated a significant reduction of the 30-day PPMI rate and new-onset LBBB. In our series, there were no differences about pre- and postdilatation, LVOT calcification, severity of aortic valve calcification, or previous conduction disturbances between groups and would not have affected our findings.

There was one acute coronary occlusion in the COVL group; however, it was not related with valve positioning. It was possibly caused by stent thrombosis, probably related to postprocedural hypotension in a patient with two long previous stents in the RCA with some distal residual plaque. Coronary access was easy, and acute closure was solved with two additional stents implantation.

Other research has shown that membranous septal (MS) length represents an anatomic surrogate of the distance between the aortic annulus and the bundle of HIS, and it is inversely related to the risk of conduction system abnormalities after TAVR [19]. Unfortunately, this information was not available in our cohort, and we could not compare these findings with our results. Moreover, our strategy would be complementary with preprocedural MS length measurements to select the limit for THV depth into the LVOT that would be easier to achieve with a better LVOT view in R-L COVL projection which is usually obtained in the right anterior oblique (RAO) view with caudal angulation. On this view, an optimal visualization of the LVOT which usually looks elongated in comparison with the LAO view has been shown using multislice CT scan images [20].

Although noncoronary and right-cusp overlap has been also suggested, we believe that a more elongated view of the LVOT and thus a better view of THV depth is achieved in R-L cusp-overlap view.

Although there are some data suggesting a higher late mortality rate after TAVR in patients with new-onset persistent LBBB, especially in those with QRS >160 milliseconds, isolated LBBB is not a current indication for PPMI and we have not used during our series. [21].

### 4.1. Study Limitations

Despite very promising results, our study has several limitations, especially all those of any observational retrospective series.

As this study was not randomized, a selection bias could be present, but somehow it may have been overcome, as two consecutive series were compared. Moreover, even in the case that the comparison between these two groups is still not appropriate, a single digit PPMI rate (6.4%) for self-expandable THV in low-intermediate volume centers sounds like a significant achievement, even possible to be improved with the evolution of the learning curve of the COVL technique.

Enrollment period was longer than expected because the number of regular cases was severely affected by COVID-19 pandemic and lockdowns in the three countries where the study was conducted.

Considering that operators' learning curve and the evolution of techniques and devices over the years may have affected PPMI implantation rate, we compared our last 101 patients with CON strategy, with the aim of minimizing this potential effect. Moreover, all procedures were performed only for the first four principal operators with significant experience using the THV used, which could have reduced operators' variability. Nevertheless, the effect of a learning curve on the results is still possible, but it is also important to consider that all patients on the COVL group were included during the learning curve of the operators with this new strategy and a low PPMI rate was achieved from the beginning of the experience and consistently sustained.

Although, implantation depth, measured by CT scan at follow-up was not available for all patients because it is not our routine clinical practice, we consider that our clinical findings related to this fact are more relevant and would overcome this potential limitation.

The decision to implant a PPM was ultimately at the discretion of the local heart team. However, except for class I indications, the threshold for choosing to implant a PPM may differ among physicians and even institutions, but the four heart teams were stable over time and may have reduced this possibility.

## 5. Conclusion

In this series, the right and left cusp-overlap view decreases the 30-day LBBB and PPMI rate without any significant MACE rate difference in comparison with the conventional 3-cusp view for TAVR implantation. Therefore, a larger, multicenter, and probably randomized clinical trial would be needed to confirm the safety and efficacy of this new implantation strategy.

## Figures and Tables

**Table 1:** Population characteristics.

	CON group, *n* = 101 (%)	COVL group, *n* = 156 (%)	*p*
Age (years)	79.8 ± 7.9	79.6 ± 7.4	0.9
Male	49 (48.5)	79 (50.6)	0.73
Hypertension	90 (89.1)	138 (88.4)	0.89
Diabetes	21 (20.8)	33 (21.1)	0.94
Dyslipidemia	69 (68.3)	107 (68.5)	0.96
Previous AMI	23 (22.7)	36 (23.1)	0.92
Previous CABG	19 (18.8)	31 (19.9)	0.83
Previous PCI	32 (31.7)	46 (29.5)	0.45
PCI before TVAR (<3 months)	22 (21.8)	31 (19.9)	0.71
Stroke	5 (4.9)	4 (2.6)	0.30
COPD	19 (18.8)	31 (18.9)	0.83
eGFR (ml/min)	60.1 ± 19.3	60.3 ± 18.9	0.82
eGFR <60 ml/min	25 (24.7)	37 (23.7)	0.64
eGFR <45 ml/min	11 (10.9)	16 (10.2)	0.87
Dialysis	3 (3)	1 (0.6)	0.14
STS	5.8 ± 2.4	5.9 ± 2.6	0.80
Prior atrial fibrillation	16 (15.8)	26 (16.7)	0.86
Prior RBBB	10 (9.9)	18 (11.5)	0.68
Prior LBBB	10 (9.9)	15 (9.6)	0.93
Prior first-degree atrioventricular block	1 (0.9)	3 (1.9)	0.55
LVEF (%)	54.8 ± 10.4	55.1 ± 10.9	0.90
LVEF <35%	11 (10.9)	19 (12.2)	0.75
Aortic valve area (mm^2^)	0.71 ± 0.19	0.72 ± 0.18	0.90
Mean gradient	40.2 ± 10.7	40.9 ± 11.2	0.87
LVOT calcification	6 (5.94)	8 (5.18)	0.77
Severity of aortic valve calcification	3231.2 ± 1040.3	3298.6 ± 916.5	0.09

**Table 2:** Procedural characteristic and 30-day outcomes.

	CON group, *n* = 101 (%)	COVL group *n* = 156 (%)	*p*
Femoral access	101	156	—
Percutaneous closure	100 (99)	156	0.21

THV evolute R/PRO™			
23	3 (3)	7 (4.5)	0.90
26	18 (17.8)	39 (25)	0.17
29	57 (56.4)	75 (48.1)	0.14
34	22 (21.8)	35 (22.4)	0.85

Predilatation	58 (57.4)	86 (55.1)	0.18
Postdilatation	25 (24.5)	34 (21.8)	0.58
Valve pop-out after delivery	—	1 (0.64)	0.42

30-day outcomes			
Death	5 (4.9)	4 (2.6)	0.3
AMI	—	1 (0.6)	0.42−
Any stroke	—	1	1
Acute coronary occlusion (stent thrombosis)	—	1 (0.6)	0.42
Major bleeding	2 (2)	1 (0.6)	0.32
Vascular complication	2 (2)	6 (3.8)	0.40
Moderate aortic regurgitation	2 (2)	4 (2.5)	0.76
Severe aortic regurgitation	—	—	—
PPMI	18 (17.8)	10 (6.4)	0.004
New-onset LBBB	13 (12.9)	9 (5.8)	0.05

Hospital stay (days)	2.9 ± 1.1	2.7 ± 1.1	0.30

## Data Availability

The data used to support the findings of this study are available from the corresponding author upon request.

## Abbreviations

CON: Conventional 3-cusp coplanar view
COVL: Right/left cusp-overlap view
LBBB: New-onset left bundle branch block
LVEF: Left ventricle ejection fraction
MACCE: Major cardiac and cerebrovascular adverse events
PPMI: Permanent pacemaker implantation
RBBB: Right bundle branch block
SAVR: Surgical aortic valve replacement
TAVR: Transcatheter aortic valve replacement
THV: Transcatheter heart valve.
